# Synthesis with control of DNA nanoflowers towards biomedical applications

**DOI:** 10.1016/j.mtbio.2025.101886

**Published:** 2025-05-21

**Authors:** Nsolo M. Maarifa, Fahim El-Kassim M. Issimail, Jian He, Xingyi Ma

**Affiliations:** a School of Biomedical Engineering, School of Science & School of Marine Science and Technology, Harbin Institute of Technology, Shenzhen, 518055, Guangdong, China; b Biosen International and Briteley Institute of Life Sciences, 264600, Shandong, China; c School of Instrument and Electronics, North University of China, Taiyuan, 030051, China

**Keywords:** DNA nanoflowers, DNA nanotechnology, Rolling circle amplification, Designability, Biosensing

## Abstract

Along with the advancement of DNA nanotechnology, DNA nanomaterials have been extensively explored and applied in the biomedical field. DNA nanoflowers (DNFs), characterized as flower-shaped nanocrystals, have attracted notable interest in the biomedical field because of their large surface area relative to volume and significant surface roughness; enabling high loading capacity. Due to their unique sequence programmability, function designability, and biocompatibility, DNFs have increasingly been researched in biosensing, bioimaging and, drug delivery and therapy. Self-assembly of DNFs enables them to maintain DNA stability and provides additional functions of metal ions, which are difficult to obtain through conventional methods. However, some challenges must be addressed, such as the interaction between DNA and inorganic material and controlling the structural features like petals and DNA sequence. In this review, we discussed the designability of DNFs, we subsequently discuss the synthetic methods and controllable parameters. Then, we enumerate recent applications of DNFs in biosensing, bioimaging, drug delivery and therapy and, templating novel functional materials. Finally, we provide conclusion remarks and perspectives for future directions.

## Highlights

• DNA nanoflowers are explored as an alternative totemplating functional materials.
• Analysis of growth/morphology control strategies, including time, pH, metal ions, and enzyme activity.
• Key applications of DNFs, including biosensing, bioimaging, drug delivery, therapeutics, and material templating.
• Future perspectives and current challenges in the synthesis, control, and applications of DNFs.

## Graphical abstract

**Figure:**
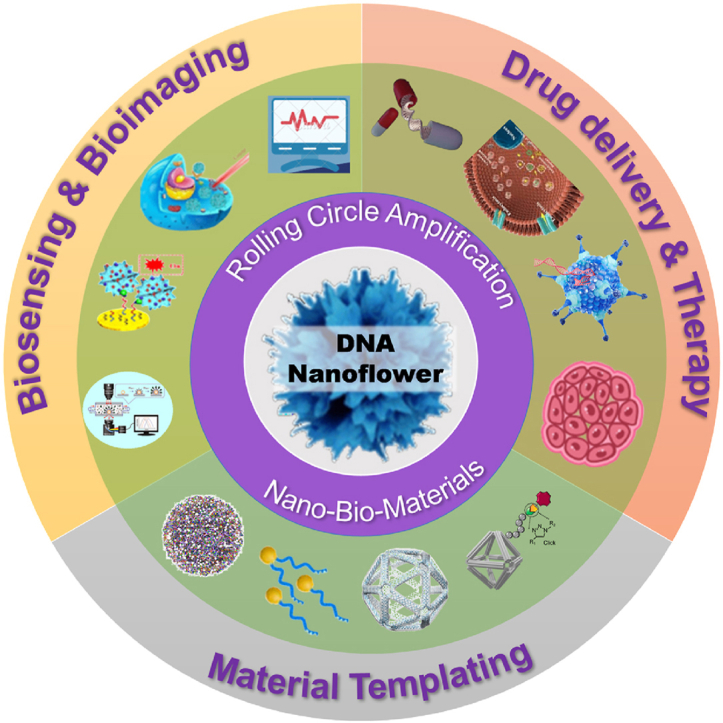

## 1 Introduction

**Fig. 1:**
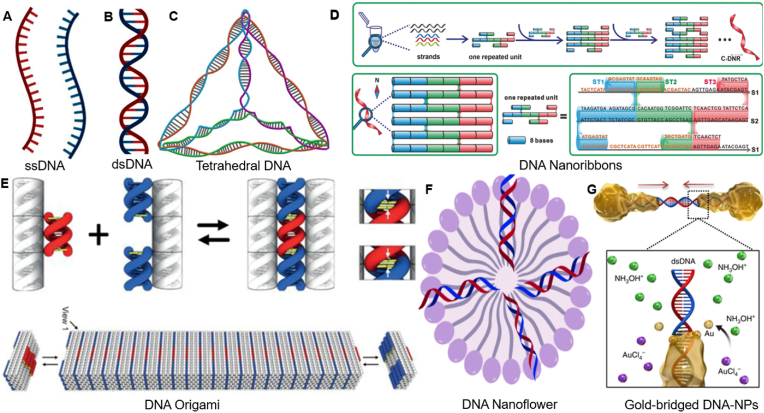
Illustration of different designable DNA nanostructures. A. Single-stranded DNA. B. Double-stranded DNA [15]. Copyright 2025, Biochemistry. C. Tetrahedral DNA assembled from ssDNA [9] Copyright 2024, Journal of Drug Delivery and Therapeutics. D. Top: Formation of DNA nanoribbons. Bottom: Schematic diagram of DNA nanoribbons, the double-helix structure is represented by a cylinder, and each small cylinder contains eight complementary base pairs [16]. Copyright 2020, Angewandte Chemie International Edition. E. DNA origami-engineered nanomaterials [17] Copyright 2022, MRS communications. F. 3D DNA nanoflower that is self-assembled through rolling circle replication [18]. Copyright 2021, Cancers. F. Shape-controlled crystallization of Au atoms to form Single gold-bridged DNA-NPs [19]. Copyright 2019, Nature Communications. (For interpretation of the references to color in this figure legend, the reader is referred to the Web version of this article.)

Recently, DNA has emerged as a highly effective material for constructing various DNA nanostructures due to their consistent and programmable structure, mechanical strength, and physicochemical robustness, utilizing Watson-Crick base pairing for regulation of intermolecular and intramolecular forces and suitability for diverse arrays of enzymes [1]. Using an uncomplicated assembly approach and design rules, researchers have developed one-dimensional (1D), two-dimensional (2D), and three-dimensional (3D) DNA nanomaterials [2] as shown in (Fig. 1). As potential therapeutic and diagnostic compounds, aptamers can be integrated into DNA nanostructures and used in the assembly procedures [[3], [4], [5]]. Generally, two major methods are used to prepare DNA nanostructures functionalized aptamers: the DNA hybridization-dependent, which is grounded on the unique specificity of A-T and G-C hydrogen-bonding, and the non-hybridization-dependent grounded on covalent bonds, electrostatic interaction, biomolecular recognition and other assembly rather than Watson-Crick base pairing approach [6,7]. These two approaches have been used to create a diverse range of DNA nanostructures notably DNA Origami [8], DNA-tetrahedron [9], DNA-nanoribbon, DNA nanoflowers (DNFs) [10], and some hydrogel structures [[11], [12], [13]] like X-DNA and Y-DNA [2,14].

Specifically, DNA nanoflowers are nanostructures formed when DNA are integrated with aptamers, they are the type of DNA hydrogel that their formation does not depend only on Watson-Crick base‐pairing interactions rather they are produced by rolling circle amplification (RCA), and efficiency packaging methods [20,21]. RCA is an isothermal signal amplification method that utilizes circular DNA template to convert DNA primer into long single-stranded DNA containing hundreds of repetitive sequences through enzymatic catalysis [22]. DNFs have found captivating applications in the biomedical field owing to their surface roughness and high surface-to-volume ratio, making them to have high loading capacity and better charge transfer [1]. Hybridization of DNFs enables them to maintain DNA stability and gives metal ions additional functionalities, which are important in diverse biomedical applications. Their high biocompatibility and biodegradability, make them suitable for delivery systems [23,24]. Recently, researchers have reported some DNAnzymes and DNFs which have been prepared as hybrid nanoflowers of biomolecule and inorganic crystals [11,[25], [26], [27]]. The DNFs presents an increased level of activity and excellent stability compared to other DNA nanomaterials [20]. This make them particularly useful for non-invasive therapeutic methods while simultaneously avoiding biological interference with treatments. They are suitable in development of therapeutic systems such as chemotherapy, gene therapy or immunotherapy used in cancer treatments. Due to their strand flexibility, more binding sites, and possessing a negative charge on their surfaces they interact easily with positive-charged particles. Thus, they can be applied as nanoscale transportation vehicles, used to transport various drugs into cellular sites and in situ encapsulations [[28], [29], [30]]. Their capability on various fluorescence signals detection, reveals them as candidates for molecular recognition in biosensors developments [1,31,32]. However, there are some challenges to be addressed like the interaction between DNA and inorganic material, the structural features of DNFs, as the petals and DNA sequence are very difficult to control [33]. Therefore, to overcome these challenges, DNFs must be designed to ensure that, they have the desirable size, morphology, properties, and functionalities.

Designability is the understanding and modification of DNA sequences, structures, and functions. It is measured by the number of sequences that can make up the structure of DNA [34]. Structures vary significantly in their designability, with few highly designable forms emerging alongside several associated sequences that are considerably above the mean. The highly designable DNA configurations consistently exhibit greater thermodynamic stability, mutational stability, rapid folding, orderly secondary structures, and symmetry tertiary arrangements.This is indicated by the quantity of sequences that the structure can support without losing thermodynamic stability [34]. These properties enable functionalization of DNA structures with numerous materials like gold nanoparticles [19], silica nanoparticles [35], polymers, and hydrogels [11,13]. The conjugation of functional materials into DNA structures contributes to new properties, resulting in diverse collective phenomena, including near-field enhancements, optical magnetism, plasmon hybridization, near-field microscopy, and biochemical sensing methods [36]. Herein, we offer a comprehensive overview of DNFs synthetic methods. Subsequently, we discusss the factors influencing the size and morphology of DNFs for tuning their biomedical applications. Finally, we provide conclusion remarks and expound on their future perspectives.

## 2 Synthesis of DNA nanoflowers

Numerous approaches have been developed for the fabrication of nanomaterials. However, bottom-up, top-down, or a combination of the two are frequently used methods [37]. In general, biomolecules containing metal-binding sites can form ionic complexes through bonding. Biomolecules like enzymes interact with copper ions and form ionic complexes due to the presence of nitrogen atoms in their amido and amino groups [38]. Their coordination drives the formation of primary nanoparticles while functioning as binding agents for petals in the DNA nanoflowers [39]. DNA has a large amount of nitrogen atoms in its backbone which enables it to be used in synthesizing DNFs through bonding with metal ions [40]. The methods employed to synthesize DNA nanoflower depend on the desired applications; for instance, rolling circle amplification (RCA), which involves mass transfer from liquid to a crystalline solid, is common for fabricating DNFs applied in drug delivery, intracellular imaging, and cell-specific targeting [39,41,42]. Similarly, the hydrothermal method uses minerals dissolved in hot water under high-pressure conditions, is commonly used for the DNFs applied to remove heavy metals from the water [26,43]. Green synthesis methods and bio-mineralization are also used to synthesize nanoflowers [44]. Following these methods, researchers have reported various DNA nanostructures including DNA hydrogels [13,45], DNAzymes [23,46,47] and DNFs [2,48,49]. The reported nanostructures possess high ability for load carrying particularly making them the preferred choices in biomedical field.

### 2.1 Rolling circle amplification (RCA)

The isothermal amplification method is employed to quickly and effectively synthesize and replicate long DNA/RNA. DNFs are generated from self-assembly condensation of a large amount of amplified DNA building blocks elongated through rolling circle amplification [50]. The advantage of the RCA-based strategy compared to conventional methods is the use of only one template strand and one primer strand [22,51]. DNFs are self-assembled via an anisotropic DNA liquid crystallization method, to circumvent the complicated sequence design required in traditional DNA nanostructures [52]. By manipulating circular template sequences, various functional DNA sequences, such as DNA aptamers, DNAzymes, and restriction enzyme sites, can be engineered into the ultralong ssDNA [40]. The designability of DNFs encompasses several key aspects. For instance, the DNA sequence design, nucleotide composition, regulation of components concentrations, and structure features of DNFs that are determined by thermodynamics and kinetics of reaction are the key in design. These factors enable the designed DNFs to perform a specific function. Template, primer, DNA ligase and its buffer, DNA polymerase and its buffer, and deoxynucleotide triphosphates (dNTPs) are important materials in RCA. Specifically, the reaction involves three basic steps. First, the template and primer are combined in ligation buffer and, the mixture is heated at 95 °C for 5 min. The mixture is then cooled down to room temperature (RT) at a controlled rate (usually 0.5 °C per minute) [20]. This facilitates the primer and template complementary bases to anneal through hybridization. The second step is the formation of circular template through ligase-mediated ligation process. The DNA ligase is added to the cooled mixture and the reaction is incubated for several hours either at RT for faster ligation or at 16 °C for slow ligation. It is recommended to incubate the reaction at low temperature for long time, as this assures the full ligation and the formation of stable ligated template [53]. The third step involves the formation of DNA nanoflower, in which DNA polymerase amplification is performed using phi29 or Bst DNA polymerase which elongate the primer to generate amplified circular DNA sequences [52,54].

**Fig. 2:**
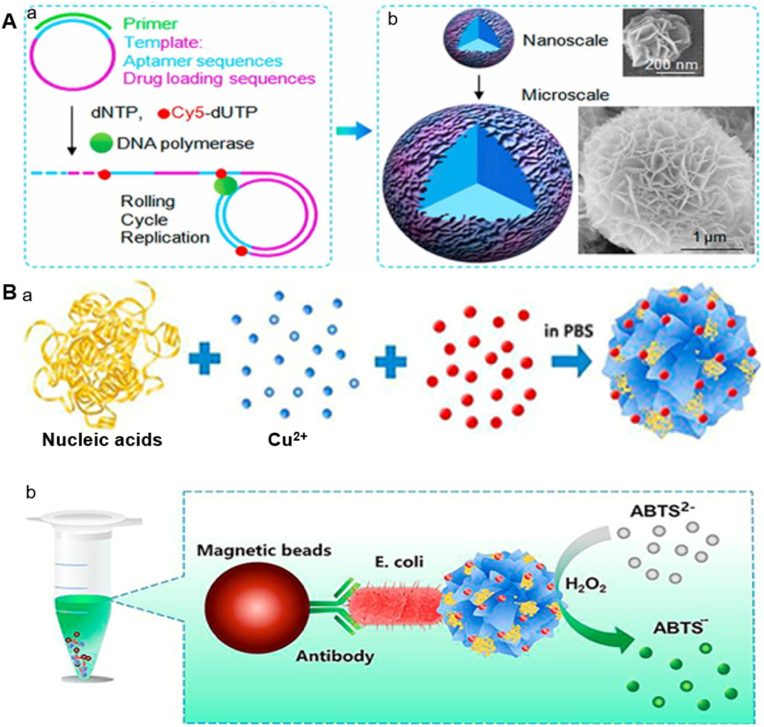
Synthesis of DNA nanoflowers. A. RCA-based assembly of DNA nanoflower. a) Modulated long DNA building block. b) Self-assembly of DNA nanoflower [4]. Copyright 2013, Journal of the American Chemical Society. B. Salt aging method a) Formation of nuclease-resistant NFs modified with hemin. b) Schematic depiction of the detection mechanism [59]. Copyright 2018, Bioorganic & Medicinal Chemistry Letters.

The elongated DNA Strands are self-assembled and hybridized leading to nucleation and growth of nanoflower complex. Metal ions such as Mg^2+^ is a crucial cofactor of polymerase in the formation of magnesium pyrophosphate (Mg_2_PPi) inorganic framework. When dNTPs molecules are enclosed into ssDNA, PPi_4_ molecules are formed as by-products leading to the generation of a large amount of PPi_4_. The produced PPi^4−^ interacts with the free Mg^2+^ to create an insoluble Mg_2_PPi that functions as the Mg-DNF framework for growth of nanocrystal (Fig. 2A). The size and morphology of DNFs can be modified through adjusting reaction time and regulating Mg^2+^ concentration [41]. Various studies reported to employ the RCA method to fabricate DNFs, hydrogels and DNAzymes for biomedical applications [11,12,51,[55], [56], [57], [58]]. Using RCA technique, Zhang et al. developed an intelligent DNA hydrogel designed for isolation of bacterial outer membrane vesicles and cancer immunotherapy [13]. Similarly, Dai et al. reported to synthesize a fluorescent copper-DNF which were self-assembled through RCA for targeting specific cells in MicroRNA imaging [39]. The RCA method has been successfully utilized in DNF synthesis, achieving significant progress in probe development. This highlights its simplicity, efficiency and versatility in the biomedical field.

### 2.2 Salt aging

Salt aging has emerged as one of the most advanced methods for the precise control of DNFs. This method, not only involves non-toxic chemicals, but also, the size and morphology of the DNFs can be precisely controlled based on the reaction parameters such as incubation time, DNA concentration, sequence type and length [60,61]. Additionally, due to the tunability of the DNFs size, often DNFs synthesized following this approach exhibit higher loading capacity [61]. In the salt aging method, DNA templates and phosphate-buffered saline (PBS) containing metal ions such as Cu^2+^ are mixed to prepare DNA-Cu_3_(PO_4_)_2_ nanoflowers complex (Fig. 2B) [62]. Typically, DNA strands of varying lengths and sequences are incubated in a PBS solution containing CuSO_4_ at RT for three days. During this process, the DNA and metal ions hybridize through the bonding of metal ions and the carboxyl groups of the DNA, leading to the formation of DNFs [63]. Based on this, Park et al. reported to synthesize an organic-inorganic hybrid NFs [62]. The size and morphology of DNFs formed were controlled through incubation time and DNA concentration. It was observed that large DNFs formed at low concentrations, and small were formed at relatively high concentrations. The effect of several DNA sequences leads to flower-like structures ranging between 20 μm and 50 μm. The method allows for DNA strands of any length and compositions, including single nucleotide, and is not limited to ssDNA or dsDNA structure and sequences, displaying its remarkable versatility. Salt aging method has since been reported in various studies, highlighting its significance in biomedical field [[64], [65], [66]].

### 2.3 Hydrothermal

The hydrothermal technique involves the thermal decomposition of a precursor metal in aqueous solutions within a closed system. In this process, the mixture of DNA solution and metal ion is placed in an autoclave or sealed vessel and heated at high temperature to promote the growth of nanostructure [67]. Under these conditions, DNA folds and interact through base pairing, as the reaction proceed the metal ion help in stabilization of DNA by reducing electrostatic interaction leading to the formation of DNFs [43]. Wang et al. used this method to prepare a novel entropy-driven multicolor DNF for rapid and accurate detection of multiple heavy metal ions in water [26]. In their study, the DNF was prepared by adding nine DNA oligonucleotides in the hybridization buffer and mixed together thoroughly. Then the mixture was heated at 90 °C for 10 min and cooled rapidly to 4 °C within 30 s, leading to the formation of DNFs. Hydrothermal method allows the control of the compositions of reaction through liquid phase or multiple chemical reactions. In addition, the morphology of the DNFs formed can be controlled by adjusting the pressure condition in either low-pressure or high-pressure depending on the vapor pressure of the main composition in the reaction.

## 3 Size and morphology control

Controlling the size and morphology of DNA nanoflowers is critical for tailoring its various applications. Through carefully manipulating different parameters, one can achieve precise control of size and morphology of DNFs for enhanced performance in biosensing and drug delivery. Thus, optimization of various parameters is crucial for successful synthesis and improvement of the functionality of nanostructures. For example, controlling temperature and pH of the reaction during synthesis of DNFs can enhance the stability of nanostructures formed as well as accelerating the rate of reaction [66,68]. The DNA template concentration, the length of sequences and the activity of enzymes significantly influence the yield of DNFs. Additionally, the morphology of DNFs depends on the type of metal ion used and its concentration. Therefore, through adjusting metal ion, uniform DNFs can be formed [69].

In summary, the size and morphology of DNFs have significant impact on their physical and chemical properties which are crucial for their applications in biosensing, bioimaging and drug delivery [41]. Specifically, by adjusting various reaction parameters, one can modify the properties of DNFs, affecting the diffusion rate, loading capacity, biocompatibility and their optical behaviors. For instance, smaller DNFs (150 nm–500 nm) are often utilized in therapy as reported elsewhere [14,23,33,63,70] because their size allows for high diffusion rate into cells. Furthermore, smaller DNFs functionalized with plasmonic nanoparticles are particularly effective in enhancing optical properties of materials. Their small size allows for easy interaction with incident light making them suitable for applications in bioimaging and plasmonic sensors. In contrast larger nanoflowers posses high loading capacity due to their larger surface areas making them suitable for carrying high amount of drugs [[71], [72], [73]].

### 3.1 Reaction time

The duration of the reaction is among the factors that influence the size and morphology of DNFs. Precisely, with a short reaction time, the size of DNF is small, and its morphology is not well defined, but as the time of reaction increases, well-defined structure and size can be observed. Most studies reported a reaction time ranging from 2 h to 30 h in RCA [22,50,74] and 3 h–72 h in the aging method [62,65,66,75] as optimal for the formation of DNFs with various sizes and morphologies, while hydrothermal only need 5–15 min to complete the reaction. For instance, Lv et al. investigated the effect of time in DNFs formation by performing the RCA reaction for 30 h [50]. The results showed that, the flower-like structures started forming after 2 h of the reaction, and the size and morphology changed from 150 nm in 6 h to 4 μm in 30 h. This confirms that the strong and highly concentrated DNFs are produced by extending the reaction time, and their size is strongly dependent on incubation time. Production of DNFs can be monitored in desired size depending on the applications, For instance to synthesize DNF for therapy small time duration in the reaction is most preferable because the size of DNF produced will have the required properties.

### 3.2 Metal ions

The metal ions used in the preparation of DNFs can significantly affect their size and morphology. Metal ions such as Mg^2+^, Mn^2+^, Zn^2+^, and Co^2+^ along with DNA polymerase as cofactors, play a vital role in DNFs formation [53,76]. They are essential in the primary crystal nucleation step and metal-enzyme coordination to produce DNFs [66,77]. Metal ion concentration affect the features and structure of DNFs. As such, higher metal ion concentration leads to an increased nucleation rate, resulting in greater quantity of particles with a reduced average diameter. Chen et al. prepared DNFs using the RCA method and investigated the effect of metal ion concentration on the size and loading capacity of DNFs [78]. A change in the Mg^2+^ concentration was observed to influence the particle size and DNA loading ability. At 10 mM concentrations of Mg^2+^, the particles were observed to have minimal quantity of DNA. However as the Mg^2+^ concentration rose from 10 mM to 25 mM, the level of DNA loading progressively increased, accompanied by a corresponding reduction in the size of the particles from 3 mm to 0.8 mm. DNA polymerase activity changes when different types of metal ions are used in the reaction and may cause the reaction to collapse. Somturk et al. investigated the effect of metal ions on the size and structure of DNFs, and they synthesized hybrid nanoflowers using a chloroperoxidase enzyme with four different metals: cobalt, manganese, calcium, and zinc [79]. While structures formed with Mn^2+^ and Ca^2+^ ions were similar, those formed with Zn^2+^ and Co^2+^ ions exhibit a distinct differences.These results witnessed the influence of different metal ions in the structure of DNFs.

### 3.3 The pH of reaction

The reaction pH is another critical factor influencing the size and morphology of DNFs. It significantly affects the surface charge of the DNA, which in turn determines its affinity and interaction with metal phosphate nanocrystals [68]. This relationship highlights the importance of pH in designing and optimizing DNFs. At lower pH values, the formation of DNFs is hindered by the repulsive force between biomolecules and metal phosphates in comparison to higher pH values. Noma et al. investigated pH's effect on hybrid inorganic nanoflowers (HNFs) and found no flower-shaped structures with a pH of 5 instead HNFs were formed at the pH range of 6–9 [80]. The activity and morphology of HNFs were greatly affected by the pH of the mixture.

### 3.4 DNA template concentration

The formation of DNFs is highly influenced by the concentration of DNA circular template. The DNFs formed at different template concentration are likely to posses different characteristics in both size, morphology and functionality [25]. Critical concentration of DNA template is the concentration that has ability to maintain the local concentration of the reaction. According to hypothesis, in RCA the local concentration of the reaction is increased with the increase of DNA circular template concentration under optimal conditions, the formation of DNFs depends on the DNA critical concentration [4,39]. To verify this hypothesis, Park et al. investigated the effect of DNA concentration on the morphology and size of DNFs [62]. The reported results indicated DNF produced at low concentrations was larger than those produced at high concentrations, the size and morphology of DNFs was mainly influenced by DNA concentration. In addition, He et al. reported to synthesis DNF and investigated the effect of circular template concentration [24]. In their study, the higher photocatalytic activity of the DNFs was observed when the concentration of circular DNA was 0.5 μM while the lower observed at 1.0 μM. The scanning electron microscope (SEM) results revealed the more compact, denser and spherical DNF at concentration of 0.5 μM while those at 1.0 μM were blurred and non-uniform. Furthermore, Zhu et al. investigated the optimal DNA concentration for the formation of DNF [4], in their work they used different template concentrations (10, 30, 100, and 200 nM) for synthesis of DNF. Their results indicated that the shape of DNF was not observe until the concentration increased up to 100 nM. The DNFs within concentration range of 100 nM–200 nM were not monodispersed compared to those with DNA concentration of 300 nM. The result confirms the hypothesis, that DNF is assembled when the local DNA concentration achieved a critical concentration. By varrying the DNA template concentrations researchers can control the formation of DNFs. Thus, DNA template concentration is highly important for nucleation, growth, and assembly of DNFs complex.

### 3.5 Enzyme activity

During preparation of DNFs via RCA method, DNA polymerase perform a role of replication, folding and stabilization of the DNFs. Specifically, through amplification of the short ssDNA sequences, they are transformed into long strands DNA which in turn fold and form DNFs. Thus, DNA polymerase activity has a significant effect on size and morphology of the final DNF [24]. Additionally, the activity of DNA polymerase depends on the type and concentration in the reaction. For instance, RCA carried out using Ф-29 DNA polymerase and that with Bst DNA polymerase will have different NFs even if they are synthesized under similar reaction conditions. To study the effect of DNA polymerase concentration, He et al. investigated the effect of the concentration of Ф-29 DNA polymerase in DNF formation through measuring the photocatalytic activity of the DNF [24]. In their work, the optimal concentration of DNA polymerase was found to be 0.01 U/μL characterized by enhanced photocatalytic activity of DNF. Using SEM techniques they proved that, at 0.01 U/μL the strcture of DNFs formed was clear and monodispersed. More over, Baker et el. investigated the effect of types of polymerase in DNFs formation [69]. In their study they compared the products formed when different DNA polymerase (Ф-29 and Bst) were used in the production of DNFs. It was observed that, the activities of the two DNA polymerase (Ф-29 and Bst) produced two different results regardless of all other conditions to be the same. By using Bst DNA polymerase the flower-shaped structures of DNFs were generated at 15 mM, while that of Ф-29 DNA polymerase generated at 10 mM. This confirm that, the activity of DNA polymerase has significant impact in the synthesis of DNFs. Table 1 lists out some of the latest studies reporting the synthesis with control of DNFs and their biomedical applications.

**Table 1:** latest studies reporting the synthesis with control of DNFs and their biomedical applications.

DNA nanoflower	Size (μm)	Detection limit/Loading capacity	Application	Ref
Multi-Aptamer-HDFs	0.931 ± 0.082	–	Rapid diagnosis in cytology specimens	[1]
Fluorescent Copper-DNA Nanoflowers	0.18–1.45	373-miRNA	Cell-Specific-Target MicroRNA Imaging	[39]
DNAzyme-Based nanoflowers	0.8	69.21 %	Reversing multidrug resistance in breast cancer	[46]
Bifunctional DNA nanoflowers	–	1.12 fg/mL	Detect ochratoxin A (OTA)	[81]
SA-HRP-Cu_3_(PO_4_)_2_ hybrid nanoflowers	5	78 pg/mL	Detection of disease-related biomarker	[82]
Programmed DNA nanosponges	0.3	–	Enhanced photodynamic therapy	[63]
Artificial base-incorporated DNA nanoflowers	0.05–1.0	–	Cancer-targeting	[33]
DNF@AuNCs	–	7 pg/mL	Detection of AFB1	[10]
DNAzyme nanoflowers	0.1–0.6	–	Enhanced cancer therapy	[23]
Magnetic RNA nanoflowers	–	50 HeLa cells	Combined drug delivery and targeted therapy	[71]
DNA-Inorganic hybrid Nanoflowers	14 ± 1.5	0.41 nM	Detection of miRNA	[83]
Artificial base-incorporated DNFs	0.05–1.0	–	Cancer-targeting and chemo-dynamic therapy	[54]
DNA-Cu_3_(PO_4_)_2_ hybrid NFs	–	0.93–2.97 μg/mL	Electrochemical detection	[49]
Oxygen-producing and pH-responsive targeted DNA nanoflowers	0.396	73.24 %	Enhanced chemo-sonodynamic therapy of lung cancer	[42]
Multicolor DNFs	0.25–2.0	–	Cellular imaging and drug delivery	[84]
DNA Nanocomplex	0.255	–	Gene/Chemo-dynamic therapy	[47]
Self-Assembled DNA/SG-I Nanoflower	0.2–2.2	0.5–80.0 ng/mL	Detection of disease-related markers	[24]
Polymer-DNA assembled nanoflower	0.92 ± 0.29	85.6 % ± 5.0 %	Targeted delivery of dolastatin	[73]
DNA flower structure	0.3	–	Targeted treatment of infectious diseases	[70]
HRP-loaded DNA flowers	–	0.001–100 ngmL^−1^	Detection of protein	[85]
Multifunctional DNF	∼0.2	71.4 %	Cell targeting and drug delivery	[86]
Enzyme-activated DNF	0.58	0.27 ppt	Ultrasensitive detection of AFB1	[31]
Sgc8-NFs-Fc	0.050–1.0	0.3 μM/23NC	Cancer targeting	[33]
Multifunctional DNF	0.18	–	Photodynamic cancer therapy in vivo	[20]
Noncanonical DNA nanoflower	∼0.2	–	Biomedical application	[4]
ZnO/Amp@DNFs	∼0.13	25.9 %	MRSA Keratitis target treatment	[21]
DNF/Cas9/sgRNA	0.46	100 nM	Cytosolic protein delivery and enhanced genome editing	[72]
Antisense DNA microsponge particles	0.2–2.0	–	Cancer therapeutics delivery	[87]
DNA immune-nanoflowers	0.3	–	CpG delivery; nuclease degradation protection	[88]
CnDNF aptasensor	0.35–0.45	85 %	Intracellular ratiometric aptasensing	[48]
Aptamer-functionalized NFs	0.77	118.7 %	Synergetic obesity therapy	[29]
Multivalent DNA Flowers	∼2.3	2.0 x10^5^particles/μL	Isolation of T-EVs	[3]
Multifunctional DNFs	0.4	1/40 Ce6-cDNA	Low-dose photodynamic therapy	[89]
ZnO-loaded DNAzyme nanosponges	0.22	–	Anticancer drug delivery and release	[28]
Rutin@ DF-RVG29	0.18	95 %	Synergistic therapy for Alzheimer's disease	[90]

## 4 Applications

### 4.1 Bioimaging and biosensing

**Fig. 3:**
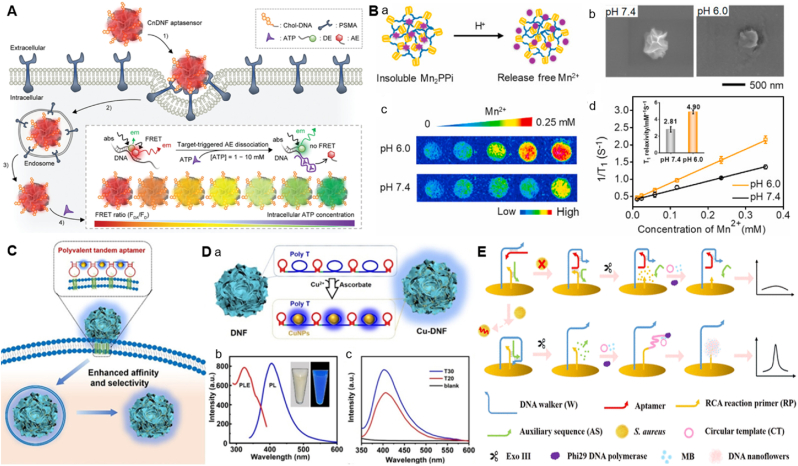
Application of DNFs in biosensing and bioimaging. A. Schematic depiction of the functioning principle of a cholesterol-decorated DNA nanoflower intracellular ratiometric aptasensor [48]. Copyright 2021, Advanced materials. B. Magnetic resonance imaging of DMNF. a) Schematic showing the acid-triggered release of Mn^2+^ b) SEM images at pH 6.0 and 7.4 c) T1-weighted images at varying Mn^2+^ concentrations under pH 6.0 and 7.4, d) Plot of Δ1/T1 versus Mn^2+^ concentration for untreated and treated DMNF [52]. Copyright 2021, Biomaterials. C. Illustration of Cu-DNF's cellular targeting and endocytic effects mechanism [39]. Copyright 2025, ACS Applied Bio Materials. D. Optical properties and stability of Cu-DNF a) Schematic outlining the synthesis strategy Cu-DNF, b) Fluorescence excitation and emission spectra of Cu-DNF and, c) Fluorescence spectra of Cu-DNF templated by poly T strands of different lengths [39]. Copyright 2025, ACS Applied Bio Materials. E. Illustration of the biosensor for *S. aureus* utilizing DNA walker and DNFs [37]. Copyright 2021, ACS Applied Materials & Interfaces.

DNA nanoflowers have been revealed as promising DNA nanomaterials for bioimaging and biosensing owing to their abundant loading capacity and spatial arrangement [33]. Due to the relaxed and permeable structures, it is easy to load many signal sensors in nanocarriers [90]. For DNFs to target the small molecules the organization of nanocarriers is done by incorporating aptamer sequence in the template or through chemical modification. With their 3D structures, DNFs are capable of cell membrane penetration without additional transfection factors. Providing good biocompatibility of DNFs for intracellular imaging [38]. DNFs are functional materials formed through the process known as biomineralization, in which organic matrices mediate the formation of inorganic minerals. In biomineralization the organic-inorganic hybrid nanoflowers are formed using living organisms in which proteins and metal are mixed in a solution and the reaction is running under mild conditions [52,91]. The mechanism involves the formation of DNA template that binds with metal ions, facilitating nucleation and the formation of complex which grow to DNA hybrid nanoflower. The formation of DNFs by biomineralization can be controlled at all levels, including the crystal composition, structure, nucleation, growth and morphology. Based on this, Kim et al. reported the design of a cholesterol-decorated DNF for prostate cancer cells targeting as well as intracellular adenosine triphosphate (ATP) imaging (Fig. 3A) [48]. The prepared DNF was delivered through the prostate-specific membrane antigen, binding aptamers with prostate-specific membrane antigen in the cellular membrane and following cellular uptake [48]. In their design the DNA template was incorporated with three different functional sequences, a prostate-specific membrane antigen (PSMA) binding aptamer, an ATP-binding aptamer, and a cholesterol-binding motif. The results confirmed that particle size and FRET signals of aptasensor can be easily modulated by using chol-DNA strand. Notably, the probe exhibited the enhanced cellular internalization in PSMA-rich androgen-dependent prostate cancer cell line (LNCaP) cells. Additionally, Zhao et al. presented a DNA-Mn nanoflower (DMNF) for MR imaging activated by a tumor site where the manganese ions facilitated enzymatic biomineralization of DNF (Fig. 3B) [52]. The signal probe was integrated with a template made of a long DNA strand through the growth of Mn_2_PPi. To achieve a large amount of cellular uptake and tumor targeting, the DNA template incorporated with the tumor specific aptamer sequence was used. The Mn^2+^ was examined as the cofactor of a DNA polymerase for the DNA strands extension; the acidic condition at tumor sites led to the collapse of DMNF morphology and the release of Mn^2+^ resulted in an improved T1-weighted MRI effect for the imaging of DMNF-treated tumor areas [52]. Similarly, combining the properties of DNA nanoflowers and copper nanoparticles, Dai et el. developed a versatile fluorescent copper nanoparticles DNA nanoflower (DNF-Cu) [39] for cell-specific-target miRNA imaging (Fig. 3C). They employed RCA to generate tandem poly T, polyvalent CD63 aptamers domain and anchoring. The tandem poly T domains efficiently chelated copper ions to promote the formation of copper nanoparticles. The polyvalent CD63 aptamer domains was used for selectivity and stability of cell expressions improving tumor uptake and facilitating endocytosis escape from lysosomes. The anchoring domains allowed integration with functional nucleic acids. The synthesized Cu-DNF demonstrated superior fluorescence properties (Fig. 3D), effectively addressing the constraints associated with single DNA or metal nanomaterials. Furthermore, Yan et al. used a functional DNA aptamer concept to design an improved and robust innovative electrochemiluminescence bioaptasensor (IEC-BA) for highly sensitive detection of AFB1 utilizing synergistic interaction and enzyme-driven programmable assembled 3D DNF [31]. The biosensor was developed via dual amplification, and they found that it has a high loading ability and sensitivity, and its limit of detection was recorded as 0.27 ppt. Also, Liu. et al. reported to synthesize micrometer-sized DNFs and enforced it as a biological recognition component to make a paper biosensor [92]. The DNF was firmly attached to the paper surface via physical adsorption. Gold nanoparticles modified with complementary sequences were used as printed calligraphy's color translators. The high surface density favored DNFs to exhibit increased withstand to nuclease deterioration and limited unspecified DNA adsorption [93].

Moreover, Cai. et al. employed DNA nanoflowers initiated by a DNA walker to construct an electrochemical biosensor aimed at identifying *Staphylococcus aureus* [37]. In their design, a DNA walker was incorporated to periodically traverse the electrode surface facilitating the release of primers for RCA and enabling amplification of signal (Fig. 3E). The transfer of electrons to the surface of the electrode was improved by the combination of DNFs and a significant amount of electroactive methylene blue (MB). The biosensor successfully differentiated *S. aureus* from other nontarget bacteria in the mixture, demonstrating high specificity and excellent resistance to interference with the detection limit of 9 CFU/mL. Apart from fluorescence imaging, there are other clinical imaging tools with enhanced resolution and penetration capability like magnetic resonance imaging (MRI) and computed tomography (CT). These studies shows that, DNFs are highly functional nano-bio-materials, they can be modified with multiple functionality elements to develop bioimaging and biosensing tools.

### 4.2 Drug delivery and therapy

Due to their adjustable size, simple design, and durability of enzymatic degradation, DNFs attracted many researchers to integrate them with drugs and imaging agents. The DNFs have numerous advantages as cargo carriers and have high stability with adequate drug delivery [94]. Besides DNFs being used in drug delivery systems, they also apply in therapeutic activities. Chemotherapy, which relies on an assortment of chemotherapeutic drugs [76]; gene therapy, which involves introducing new copies of multifunctional gene or replacing a defective or missing gene in a patient's cells with a healthy version of that gene and immunotherapy which aims at regulating and killing cancer cells, and controlling the patient's immune responses, are among the potential significant therapeutic applications in which DNFs have been deployed [95]. Among the challenges in treatment of cancer and cell stimuli sensitivity is Multidrug resistance (MDR), which is triggered by drug efflux from cancer cells; DNFs are capable of controlling and can be used for drug delivery to chemosensitive and MDR tumor cells that causes MDR in cancer cells [71]. The drug loading capacity and stability, even at pH 7.4 in an acidic environment, make DNA nanoflowers a good drug delivery agent, mostly occurring in acidic environments. Apart from the structure, which causes toxicity in targeted MDR and chemotherapy-sensitive cells while reducing side effects [96], additionally DNFs exhibit excellent biocompatibility and biodegradability, making them suitable, for drug delivery and therapeutics.

**Fig. 4:**
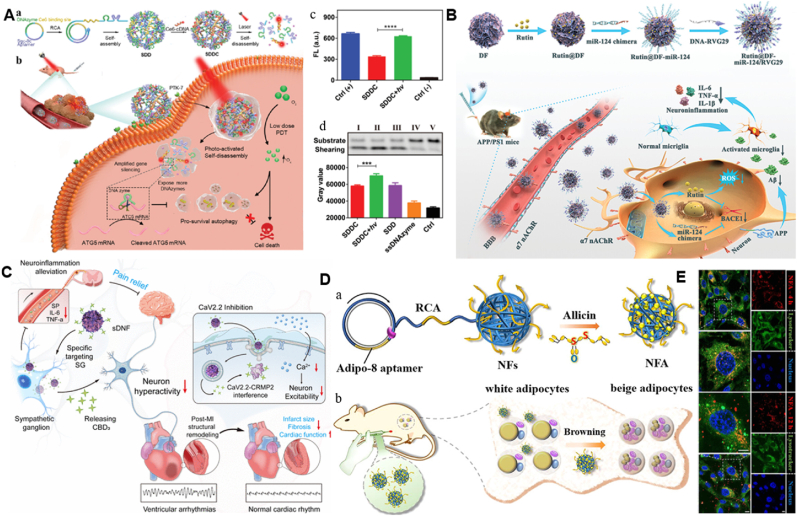
Application of DNFs for Drug delivery and therapy. A. Photonic self-disassembly of multifunctional DNF for low-dose photodynamic treatment a) DNFs are generated through RCA and incorporating Ce6 on SDD through sequence hybridization. b) Targeting of the tumor site and tumor cells uptake by SDDC through PTK-7 receptor-activated [89]. Copyright 2021, Small. B. Diagram for the development of Rutin@DF-miR-124/RVG29 (RDMR) nanosystem and its use in Alzheimer's disease therapy [90]. Copyright, 2022 Small. C. Illustration of neuron-targeting DNA nanoflowers used in treating malignant ventricular arrhythmias and cardiac remodeling [97]. Copyright 2025, ACS Nano. D. Illustration of an effective obesity management strategy through targeted activation of G4-mediated UCP1 expression. (a) Representation of preparation of aptamer-conjugated drug delivery system. (b) The NFA interact with the target receptor on the cellular membrane. E. Representative confocal microscopy images of cellular internalization [29]. Copyright 2022, ACS Nano.

To date, numerous studies have reported the uses of DNA nanoflowers in drug delivery and therapeutics to authenticate the exploitation of DNFs in drug delivery systems and therapeutics. Shi et al. reported to develope an effective autophagy blocker, which was a multifunctional DNF consisting of aptamers for targeting tumors and DNAzymes for silencing autophagy-related genes, with surface modification of low-dose photosensitizer (Ce6) [89]. They engineered a photoactivated self-destructing DNF, which facilitated a spatial-temporal release of tumor-targeted cargo (Fig. 4A). The DNFs incorporate DNA aptamer used to target tumor cells, a DNAzyme for silencing autophagy-related gene, and a sequence that hybridizes with Ce6-labeled cDNA; Sgc8c–DNAzyme/DNF-Ce6), known as SDDC. The self-destruction of DNF was triggered by the reactive oxygen species (ROS), produced during photodynamic therapy (PDT) process, thereby offering an easy method to release the light-activated DNAzymes from DNFs. It was observed that SDDC target tumor cells promote enhanced autophagy suppression and efficiently improve tumor therapy at low doses. The designed DNF suppresses tumor progression in vivo with an extremely minimal injection dose of Ce6 reflecting an optimistic approach to cancer treatment.

In addition, Ouyang et al. fabricated a DNA nanoflowers with miR-124 based delivery system for targeting Alzheimer's disease (AD) therapy, where they created a miR-124 chimera framework featuring a toehold sequence, which facilitated easy loading of DNFs [90]. To achieve blood-brain barrier (BBB) penetration and targeting of neuronal cells, they decorated the surface of DNFs utilizing a DNA-functionalized RVG29 peptide motif, capable of specific binding to α7 nicotinic acetylcholine receptor (α7 nAChR). This receptor shows a high expression level on the surface of BBB and neuronal cells (Fig. 4B). They also incorporated Rutin in DNFs via π-π stacking, to improve the effective reactive oxygen species (ROS) scavenging activity and support the reduction of Aβ generation and neuroinflammation both in vitro and in vivo [90]. Consequently, they attained a remarkable therapeutic effectiveness of AD disease treatment in APP/PS1 mouse models demonstrating a high biosafety. Recently Qiao et al. in the effort of combating myocardial infarction and neuropathic pain (Fig. 4C) used DNF to develop a peptide mediated smart DNA nanoflower (sDNF) [97]. The sDNF was equipped with neuron targeting aptamers used for selective binding and cellular uptake, then Ca^2+^ channel-binding domain 3 (CBD3) was incorporated to interfere with CaV2.2 membrane trafficking. To allow the responsive release of CBD3 they hybridized CBD3-DNA bioconjugate in situ. Their results showed that, CBD3 peptide alleviate neuropathic pains. Similarly, Chen et al*.* used DNFs to develop the aptamer-conjugated dual-drug delivery platform for combined obesity therapy [29]. They conjugated adipo-8 aptamer and loaded allicin through RCA method to form a combined adipocyte-targeted, and used it to secure allicin from adsorption (Fig. 4D). The conjugated adipo-8 aptamer displayed the notable ability to contain, convey, and release a molecular substance into white adipose tissue. To investigate DNF cellular uptake ability they used Lysotracker Green to specifically label the cell lysosomes. Confocal microscopy images (Fig. 4E) revealed that, DNF was captured in lysosomes. The platform was successfully employed to enclose, deliver, and discharge biomolecules, significantly improving the biological activity of the allicin. Basically, the designable and controlled synthesis of a regulated delivery system is key for enhanced DNF efficacy.

### 4.3 Templating novel functional materials

**Fig. 5:**
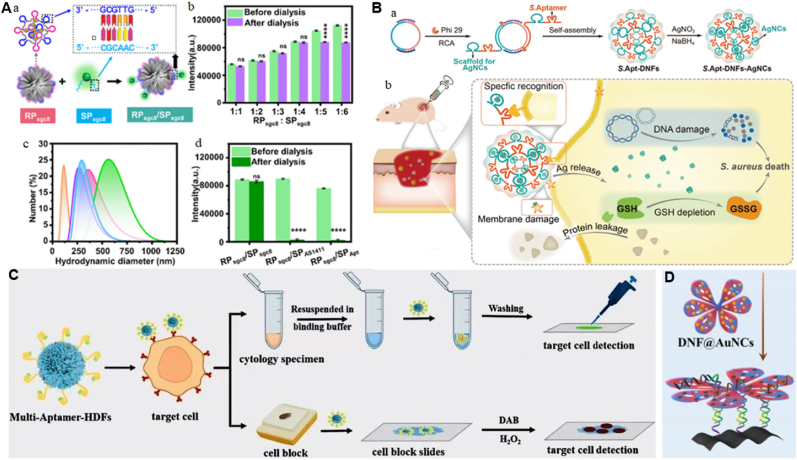
Application of DNA nanoflower in templating novel functional materials. A. antibacterial sustained delivery system through RCA. a) Construction of multifunctional S.Apt-DNFs-AgNCs. b) Antibacterial function of S.Apt-DNFs-AgNCs for treatment of wounds [98]. Copyright 2024, Advanced Healthcare Materials. B. Construction and validation of the RP_sgc8_ and SP_sgc8_ hybridization system. a) Illustration of one-step construction of the RP_sgc8_ and SP_sgc8_ hybridization system b) Fluorescence intensity of the RP_sgc8_ and SP_sgc8_ hybridization system c) Hydrodynamic diameter and d) fluorescence intensity of the RP_sgc8_ and paired/unpaired SP hybridization system [5]. Copyright 2024, ACS Applied Materials & Interfaces. C. Multi-Aptamer-HDFs recognizing the target cells [1]. Copyright 2024, Microchemical Journal. D. A fluorescent based aptasensor constructed from DNF@AuNCs and (Mn-MOF) [10]. Copyright 2024, Chemical Engineering Journal.

DNA can attach the targets like metal ions, small molecules, proteins or whole live cells, viruses, and bacteria exhibiting a strong affinity and selectivity, this makes them applied in the detection of biomolecules and cancer cells [94]. The use of DNFs to template novel materials allows the control of their structures, and organization of these materials leads to the enhanced properties of DNFs. In comparison to antibodies, aptamers comprise numerous specific benefits, qualifying them as optimal candidates for biorecognition units to construct biosensors [94]. This type of nanostructure offers many advantages, including adjustable size, simple design and formation, and durability to enzymatic degradation. Aptamers sequences could be integrated into the DNA nanoflowers to increase their functionalities. By applying the aptamer concept, Yu et al. utilized DNA nanoflower to create an RCA-sustained antibacterial delivery system with remarkable biocompatibility and two functionalities [98]. The system includes sequential tandem of silver nanoclusters (AgNCs) for killing bacteria and multivalent aptamers for enhanced antibacterial therapy (Fig. 5A). As a flexible and cost-effective method, for integrating multiple aptamers, the platform demonstrated high binding affinity and specificity for targeting pathogens, ensuring precise antibacterial delivery. Also, Guo et al. created a nano platform merging a nucleic acid aptamer and DNA nanotechnology for the imaging of living cells [5]. Recognition probes (RPs) based on aptamer were synthesized using the RCA technique, and subsequently self-assembled into DNFs encased by an aptamer. The signal probes (SPs) were acquired by attaching multicolor-emitting carbon quantum dots to RP complementary oligonucleotides. Recognition probes and Signal Probes were paired through base pairing to produce imaging systems based on aptamer sgc8 and AS1411 (Fig. 5B). In addition to that, Xiang et al. integrated DNA-aptamers into biosensors by constructing the multivalent aptamer hybrid DNA flowers with fluorescence effect and efficient targeting of cancer cell [1], for detecting lung small cell cancer. The aptamer was engineered to function as the signal recognition element, featuring both fluorescent signal output and oxidation reaction for ultra-sensitive detection of cancer cells (Fig. 5C). Additionally, Niu et al. engineered a bifunctional DNF that integrated stable AuNCs nucleation sequences with molecular recognition elements [10]. The DNF was changed from 3D to 2D through in situ preparation of AuNCs. The 2D petals of DNF created binding sites of flower templates for synthesis of AuNCs and DNA recognition (Fig. 5D). The LCHA amplification was performed based on manganese metal organic framework (Mn-MOF), which enabled to achieve signal turn-off of the platform through hybridization of G-rich strands with DNF@AuNCs. The developed strategy was applied as a signal tag in the detection of AFB1. Moreover Zheng et al. by using one-pot RCA method developed multifunctional DNFs consist of DNA G-quadruplex AS1411 aptamer and called it NF@G4/Porphyrin [20]. The developed DNFs was used as drug carriers for cancer targeting and photodynamic therapy. The role of aptamer was to carry porphyrin as well as the identifier of nucleoli. And it was observed that, the NF@G4/Porphyrin is capable to be applied in vitro and vivo without any cytotoxicity side effect. They constructed a nanomaterial with high specificity and efficiency for targeted cancer treatment. Furthermore, Ran et al. developed an innovative DNA nanoflower eye-drop system to combat MRSA induced keratitis [21]. This sophisticated system termed ZnO/Amp@DNFs combined multiple functionality elements such as bacteria-targeting aptamer and an acid response DNAzyme cleaved the mecR1 gene for gene regulations, and then the DNF was loaded with ZnO and β-lactam antibiotic ampicillin for synergistic MRSA eradication. Encoded aptamer enabled the nanoflower to intensively endocytosed by bacteria, and release DNAzyme under acidic conditions is used to cleave the mecR1 gene leading to down-regulation of PBP2a, and combined with ampicillin for effective MRSA elimination. This approach demonstrated a dual action therapy. All these applications reveal that, DNF can be used as a template for synthesis and organization of novel materials like metal nanoparticles, enzymes and proteins in a nano scale by controlling their sizes, shapes and properties, which are beneficial in biosensing, drug delivery and other biomedical applications.

## 5 Conclusions and perspectives

DNA nanomaterials have received significant interest owing to their programmability, flexibility, and tunable morphology. DNFs, with their unique morphological characteristics, including large surface area, rigidity, and biodegradability, are among the most prominent DNA nanomaterials that with potential biomedical applications including diagnosis, therapy, and biocatalysis. In this review, we outlined the main synthetic methods for the synthesis with control of DNFs for various applications, including the RCA approach, salt aging, hydrothermal synthesis, and the biomineralization method. We then focused on the synthesis factors for the precise control of size and morphology. Specifically, we highlighted the significant role of reaction parameters such as reaction time, type and concentration of metal ions, reaction pH, and enzymatic activity on the final morphology of the DNFs, thus, the application performance. Furthermore, we turned to the various applications of DNFs, focusing on its uses in bioimaging and biosensing as a diagnostic agent, in drug delivery and therapy as a drug carrier and therapeutic agent, and templating material for novel functional materials.

Besides their morphological tunability through such methods, owing to their ease of functionalization, various DNFs materials with advanced applications have been successfully achieved. Specifically, DNFs possess several advantages over other DNA nanostructures such as DNA origami. for instance, due to their dense packaging and amplified structure, DNFs are more stable under thermal conditions. Additionally, unlike other DNA nanostructures depending on Watson-Crick base pairing, DNFs can be readily synthesized using simple methods such as RCA and salt aging which increases their yield during synthesis. Furthermore, due to their flexible morphology, they exhibit higher cell uptake with transfection agents compared to other types of DNA nanomaterial. Lastly, DNFs enable the functionalization of multiple agents through programmability than other types of DNA nanostructures. However, DNFs also faces certain limitations with regard to other nanomaterials. For instance, compared to DNA origami, DNFs purification faces severe limitations due to its lack of structural integrity. Also, regarding control of size and shape during synthesis, DNFs faces challenges as they do not directly rely on Watson-Crick base pairing that limit their applications in fields requiring specific size or shape. For instance, the combination of DNF and other biomolecules for bioimaging or cancer therapy is routinely achieved with greater yield. DNF/RNA is one of the most commonly synthesized complexes owing to the flexibility of sequence designability and hydrogen bonding between nucleic acids. In addition, the functionalization of DNFs with inorganic materials such as AuNPs has also demonstrated significant promise in the field of plasmonic and fluorescent biosensing.

Despite the recent progress in the synthesis and application of DNFs, there are still persistent challenges that need to be urgently addressed. First, the structure-relationship of DNFs remains to be clearly elucidated. As several of the applications of nanomaterials such as plasmonics and drug delivery are size dependent, deeper understanding of this relationship may allow researchers to address the current limitations of DNFs in sensing and therapy. For instance, for the fabrication of plasmonic NP, the size of the DNFs is required to fall within a specific size range (lower than the wavelength of the incident light) for effective light scattering. Likewise, larger DNF sizes may influence the pharmacokinetics of the DNF/RNA or DNF/drug complex leading to accumulation at specific sites. Therefore, size control is a persistent challenge urgently needing to be addressed. Second, the interaction between DNF and other materials, especially inorganic materials, still requires to be comprehensively elucidated. Often, inorganic materials require rigorous pre-treatment before they can affectively interact with DNFs which limit the application of DNFs-inorganic complexes. Similarly, another significant challenge concerns the large-scale synthesis of DNFs which is hindered by many factors such as the reaction time and the diversity of the chemical reagents required during synthesis. And lastly, the biosafety of DNF complexes has not been comprehensively investigated yet, which pauses a significant challenge regarding their applications in biomedicine.

It is anticipated that in the future, once, the mechanistic insights regarding the synthesis and functionalization of DNF are uncovered, the influence of DNF will expand to possibly addressing the most pressing question regarding the synthesis on-demand of DNFs for advanced diagnostic and therapeutic applications. Further, this could lead to DNF being deployed as biomedicine for personalized treatments. to Hence, more efforts are required to focus on the synthesis with control of DNFs. It is also suggested that integrating computational models and the current protocols may instigate the advancement of DNF to further new applications.

## CRediT authorship contribution statement

**Nsolo M. Maarifa:** Writing – review & editing, Writing – original draft, Formal analysis, Data curation, Conceptualization. **Fahim El-Kassim M. Issimail:** Writing – review & editing, Writing – original draft, Formal analysis, Data curation, Conceptualization. **Jian He:** Writing – review & editing, Supervision. **Xingyi Ma:** Writing – review & editing, Validation, Supervision, Funding acquisition, Conceptualization.

## Declaration of competing interest

The authors declare that they have no known competing financial interests or personal relationships that could have appeared to influence the work reported in this paper.

## Data Availability

No data was used for the research described in the article.
